# Shoot Meristem Function and Leaf Polarity: The Role of Class III HD–ZIP Genes

**DOI:** 10.1371/journal.pgen.0020089

**Published:** 2006-06-30

**Authors:** Mary E Byrne

**Affiliations:** Johns Hopkins University School of Medicine, United States of America

## Abstract

The shoot apical meristem comprises an organized cluster of cells with a central region population of self-maintaining stem cells providing peripheral region cells that are recruited to form differentiated lateral organs. Leaves, the principal lateral organ of the shoot, develop as polar structures typically with distinct dorsoventrality. Interdependent interactions between the meristem and developing leaf provide essential cues that serve both to maintain the meristem and to pattern dorsoventrality in the initiating leaf. A key component of both processes are the class III HD–ZIP genes. Current findings are defining the developmental role of members of this family and are identifying multiple mechanisms controlling expression of these genes.

## Introduction

**Figure 1:**
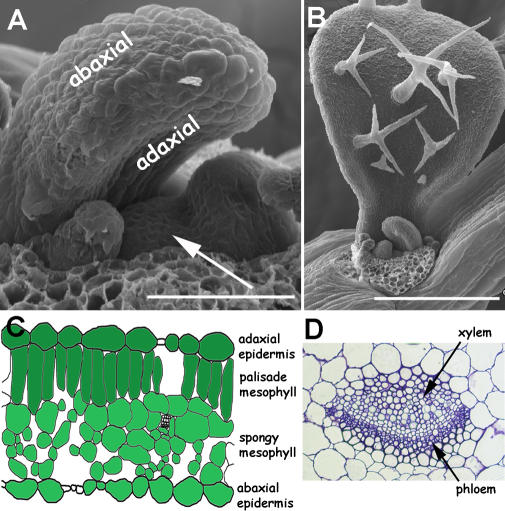
Leaves Arise on the Flanks of the Shoot Meristem (A) Vegetative apex of *Arabidopsis*. The adaxial side of the leaf is adjacent to the central shoot apical meristem, whereas the opposite, abaxial, side of the leaf is farther from the shoot meristem. Dorsoventrality is established early in development and is clearly evident in the leaf on the left, which arches over the meristem due to differential growth on each side of the leaf. Scale bar is 50 μm. (B) Developing vegetative leaf of *Arabidopsis*. Adaxial trichomes on the larger leaf are one marker distinguishing dorsoventrality. Scale bar is 250 μm. (C) Diagrammatic representation of a leaf cross section with adaxial and abaxial outer epidermal and inner mesophyll marked. (D) Cross section of leaf midvein with adaxial xylem and abaxial phloem cells marked.

A hallmark of land plant evolution has been development of the leaf. Leaves are the principal organ for capture of energy from sunlight and conversion, through photosynthesis, into organic components for growth. In angiosperms, leaves are typically planar, dorsoventrally flattened structures. Dorsoventrality is specified early in development of primordia. In the initiating leaf, the dorsal, or adaxial, side is immediately adjacent to the shoot apical meristem, whereas the ventral, or abaxial, side is farther from the shoot meristem (Figure 1A and 1B). In the mature leaf, the adaxial side is usually the upper sun-exposed side of the leaf and the abaxial side is the lower shaded side of the leaf.

A series of surgical experiments carried out in the 1950s, and elaborated upon more recently, were the initial key to mechanisms that establish leaf dorsoventral patterning [1–3]. Separation of initiating primordia from the meristem by surgical incision generated a radial, abaxial leaf. This suggested, firstly, lateral organ patterning required an interaction between the initiating organ and the shoot apical meristem and, secondly, that in the absence of this interaction loss of dorsoventrality resulted in radial organs. Thus positional information in the context of the apical meristem specifies dorsoventral patterning and development of a leaf as a planar structure.

The first hint of a molecular basis for dorsoventral patterning came from the aptly named *phantastica (phan)* mutant in *Antirrhinum* [4,5]. Severely affected leaves in *phan* mutants are abaxial and fully radial, whereas weakly affected leaves have abaxial sectors on the adaxial leaf surface surrounded by ectopic lamina. The phenotypes of *phan* are entirely consistent with a requirement for dorsoventrality in lamina development. Subsequent to the work in *Antirrhinum,* there has been a relative explosion of information in the field, with identification and description of many additional genes and potential networks that combine to pattern adaxial and abaxial fate in the leaf [6–8]. To provide a basic primer to this field, this review will focus entirely on the role of one family of genes in leaf patterning and meristem function. These genes are the Class III HD–ZIP family. Members of this gene family are both necessary and sufficient for adaxial leaf fate and they potentially represent a pivotal component for leaf patterning–shoot meristem interactions. Class III HD–ZIP genes also provide a good example of the role of miRNAs in plant development.

## Markers of Dorsoventrality

The extent to which adaxial and abaxial sides of a mature leaf can be distinguished varies between species; however, in developmental model species such as the dicot *Arabidopsis* or the monocot maize, many cell types differentiate top from bottom [9–11]. In *Arabidopsis,* epidermal cells on both sides of the leaf are jigsaw-shaped, but adaxial cells are larger and uniform in size relative to variable abaxial cell size. Trichome density is a useful marker on early juvenile leaves, where adaxial trichomes are much more frequent than abaxial trichomes. In subepidermal cell layers, closely aligned elongate palisade mesophyll cells lie juxtaposed to the adaxial epidermis. Less closely spaced, larger spongy mesophyll cells form abaxial internal tissue (Figure 1C). The palisade and spongy mesophyll tissues are optimized for light capture and gas exchange, respectively. Vasculature is also patterned in the dorsoventral dimension with xylem, the water-conducting tissue, adaxial to the organic nutrient–conducting phloem tissue (Figure 1D). Vascular bundles within the stem are also patterned with xylem more central to peripheral phloem. Conceptually, the dorsoventral vascular patterning of the leaf can be translated into a collateral central–peripheral pattern within the stem.

## Class III HD–ZIP Genes—The Arabidopsis Family

**Figure 2:**
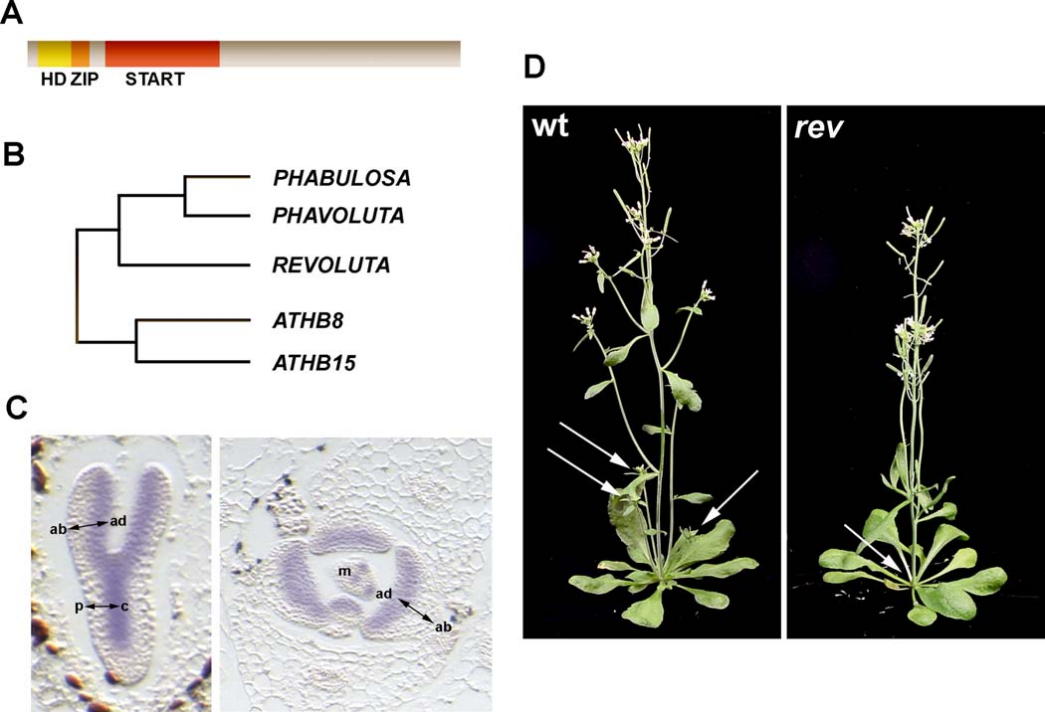
Class III HD–ZIP Genes in *Arabidopsis* (A) Class III HD–ZIP genes encode 833–852 amino acid proteins with main domains indicated; an N-terminal HD-ZIP domain, and a 213–218 amino acid START domain. (B) Relationship between five *Arabidopsis* Class III HD–ZIP genes. (C) Representation of expression pattern of *PHB* in longitudinal section of embryo (left) and transverse section of shoot apex (right). In the embryo, expression is adaxial in cotyledons and in central provasculature. In the shoot apex, expression is adaxial in developing leaves and in the meristem. ad, adaxial; ab, abaxial; p, peripheral; c, central; m, meristem (D) Phenotype of *rev* mutants. In wild-type, axillary meristems in axils of leaves give rise to lateral branches (arrows). In *rev* mutants, axillary meristems are frequently absent, and fewer or no lateral shoots are produced.

Class III HD–ZIP transcription factors have in common a homeodomain DNA binding motif and a leucine zipper dimerization motif (HD-ZIP), and are a subset of a much larger group of plant proteins that also include a sterol/lipid binding (START) domain [12,13] (Figure 2A). Although lipid ligands for a small number of START domain proteins have been identified in animals, none to date have been found for plant START proteins. There are five Class III HD–ZIP genes in *Arabidopsis,* each encoding a protein in the range of 833–852 amino acids, and sharing between 60% to 85% amino acid homology (Figure 2B). *PHABULOSA (PHB)* and *PHAVOLUTA (PHV)* are most closely related to one another, sharing 85% amino acid identity [14]. Likewise, *ATHB8* and *ATHB15* form a relatively closely related pair, sharing 75% amino acid identity [15]. *ATHB15* has also been published under the name *CORONA (CNA)* [16,17]. *REVOLUTA (REV),* in some previous work published as *INTERFASICULAR FIBERLESS1 (IFL1),* shares between 60% and 66% amino acid identity with other members of the group [18–20].

## Patterned Expression

All five Class III HD–ZIP genes in *Arabidopsis* have well-defined tissue-specific expression patterns within the embryo and shoot. There are several principal reports describing tissue specific expression patterns, as determined by in situ hybridization, for *REV* [15,18,20], *PHB* [14], and *PHV* [15]. For comparative analysis, expression patterns in embryo and shoot for all five HD–ZIP genes have also been reported [17].

*PHB* and *REV* are expressed early in embryogenesis and appear throughout the 16-cell embryo. As development proceeds, expression becomes confined to the adaxial domain of the cotyledons and the central region of the embryo, including the shoot apical meristem and provasculature (Figure 2C). The expression of *PHV* and *ATHB15* reiterate this pattern although expression initiates later and is more restricted than that of *PHB* and *REV*. The adaxial expression pattern is also in the developing organs of the shoot. However, in the shoot apical meristem *PHB* is expressed throughout very early stages of organ initiation as well as in regions that predict sites of successive organs. Discrete bands of expression extend from these presumptive organ sites to the very central region of the shoot apical meristem. The significance of this expression pattern is still to be determined. Potentially, expression of genes within these bands may contribute to coordinating central zone stem cell activity with peripheral zone lateral organ cell recruitment. In the shoot, *REV* is expressed adaxially in lateral organs, as well as in vasculature, and has a complex pattern in the shoot apical meristem, but, like *PHB,* predicts the site of organ initiation. *ATHB15* has high levels of expression in the vasculature of the shoot and in axillary and floral meristems. In contrast to other members, expression of *ATHB8* is more limited, being restricted to procambial cells of the embryo and vasculature of developing organs [17,21–23]. Some of the differences in expression patterns of these genes are consistent with differential contributions to development, as described below.

## Loss-of-Function Effects

A visible gross plant phenotype for loss-of-function mutations in Class III HD–ZIP genes has only been described for *REV,* as redundancy seems to mask the role of other family members in development [18–20,24]. *REV* mutants have a diverse range of phenotypes. One of the most prominent defects is a failure in initiation and development of secondary meristems in the axils of vegetative rosette and cauline leaves, resulting in plants with few branches (Figure 2C). Flowers display variable phenotypes and may lack organs or form only rudimentary tapered filaments. In the inflorescence stem, there is a loss of xylem and interfasicular layers interconnecting vascular bundles. Despite this loss, there is no defect in vascular dorsoventral patterning.

Loss of *PHB* and *PHV,* either as single mutants or together in a double mutant, has no evident phenotypic consequences. However, mutations in both genes enhance *rev* defects, indicating *PHB* and *PHV* are redundant with each other and also with *REV* [15,17]. Depending on the background, loss of all three genes results in either partially abaxial cotyledons without a shoot apical meristem or a single abaxial cotyledon, or, in the extreme, failure to correctly pattern embryo development. Additional loss of *ATHB15* in this background increases the frequency of the more severe phenotype. These patterning defects coincide with the expression pattern of these genes and are consistent with loss of both organ adaxial patterning and shoot central patterning within the embryo.

All HD–ZIP genes function in postembryonic development. Mutations in both *PHB* and *PHV* enhance shoot phenotypes of *rev* [17]. Like *PHB* and *PHV,* loss of either *ATHB8* or *ATHB15* or combined loss of both of these genes has no gross phenotypic effect, although vascular development is slightly perturbed in *athb15* mutants. However, mutations in *ATHB8* and *ATHB15* suppress axillary and floral meristem defects of *rev* mutants. Together, genetic interactions suggest overlapping redundant as well as competitive interactions between these genes in development [17].

## Dominant Effects

Mutations in *PHB* were first reported as temperature-sensitive, semidominant mutants with radial, adaxialized leaves and enlarged meristems [25]. Ectopic meristems surround the adaxial leaf as would be expected if adaxial fate promotes meristem formation or, alternatively, if *PHB* independently promotes meristem formation. Like *PHB*, gain-of-function mutations in *REV,* as in *rev-10d* and the allele *amphivasal vascular bundle 1 (avb1),* result in stem fasciation indicative of an increase in meristem size, adaxialized leaves, and conversion of normally collateral vasculature of the stem, where xylem is central to peripheral phloem to amphivasal vasculature with the xylem surrounding phloem [15,26]. All of these phenotypes are not always evident even with identical mutations and genetic background, suggesting that growth conditions may influence expressivity of the phenotype.

**Figure 3:**
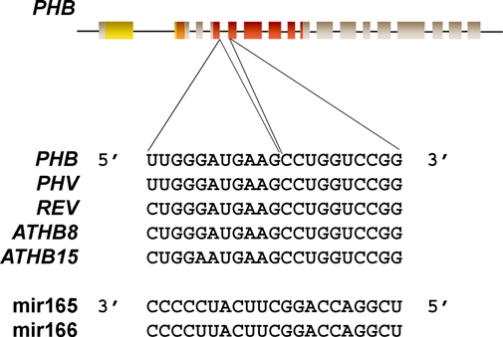
MicroRNAs and Class III HD–ZIP Target Sequences Diagrammatic representation of the *PHB* gene. Exons are represented as boxes and introns as lines. The three structural domains in the coding sequence are the HD-ZIP (yellow-orange) and the START (red) domains. Below them is the sequence of the microRNA binding site in the five Class III HD–ZIP genes and *miR165* and *miR166* sequences.

All dominant mutations in Class III HD–ZIP genes occur within a defined region around the 3′ end of the fourth exon and 5′ end of the fifth exon (Figure 3). An allelic series of *phb-d* mutants revealed either a point mutation at a splice site, resulting in a small percentage of transcripts with a short peptide sequence insertion, or point mutations resulting in an amino acid change [14]. Multiple dominant alleles of *phv* and *rev* also have single nucleotide changes within the same region as found in *phb-d* alleles [14,15,26]. However, as discussed below, changes in amino acid sequence in the dominant mutants do not appear to be significant as the site of all these mutations is within the predicted binding site for the small regulatory microRNAs, *miR165* and *miR166* [27,28].

## Regulation by microRNAs

MicroRNAs are approximately 21 nucleotides in length and are generated from longer precursor transcripts. The precursor transcripts notably form a hairpin loop structure that is recognized and cleaved into a double stranded form carrying the microRNA and a complementary sequence. Ultimately, the microRNA as a single strand is guided to the target transcript, a process involving the small RNA binding proteins of the ARGONAUTE family. Subsequently, target transcripts are either cleaved within the region binding the microRNA or are subject to translational inhibition. Much more detail on this pathway and the genes involved can be found in reviews on the subject [29–31].

The *Arabidopsis* genome encodes two copies of *miR165* and seven copies of *miR166,* which have near perfect match with a sequence conserved within the transcript of all *Arabidopsis* Class III HD–ZIP genes [27] (Figure 3). So far, regulation of Class III HD–ZIP genes by microRNAs appears to occur by transcript cleavage, as is typical for many plant genes targeted by microRNAs. Several lines of evidence support this model [15,26,32,33]. Firstly, *REV* and *PHB* cDNA constructs that alter the microRNA binding site but not the corresponding amino acid sequence induce the same phenotypes as the gain-of-function mutations in these genes. Secondly, transcript cleavage products can be generated in in vitro assays and can be detected in plant extracts. Thirdly, in *phb-1d* mutants, the mutant *PHB* transcript is ectopically expressed and occurs throughout the leaf, whereas constitutive expression of wild-type *PHB,* using a 35S promoter, does not, in general, induce a phenotype. Although constitutive expression of the Class III HD–ZIP genes does not result in a phenotype, there are two interesting exceptions. Overexpression of *PHB* did result in adaxialization of early leaves in a small proportion of transformants [33] and overexpression of *ATHB8* induces a phenotype with an increase in vascular xylem [34]. Occasional overexpression phenotypes may reflect dosage-dependent effects between microRNAs and target transcripts.

As might be expected, overexpression of microRNAs results in a reduction in Class III HD–ZIP gene transcripts. Two semidominant mutants, *meristem enlarged1 (men1)* and *jabba-1D (jab-1D),* isolated from activation tagged lines of *Arabidopsis,* have increased levels of *miR166a* and *miR166g,* respectively [35,36]. Although homozygous *men1* are seedling lethal, *men1*/+ plants have a range of phenotypes including an enlarged shoot apical meristem and slight downcurling of leaves suggesting weak abaxialization of lateral organs [35]. Meristem, leaf patterning, and vascular defects also appear in the *jab-1D* mutant, and the severity of these phenotypes is stronger in homozygous compared with heterozygous plants [36]. In both *men1/*+ and *jab-1D,* overexpression of different *miR166* genes has differential effects on expression of individual members of the Class III HD–ZIP genes. In *jab-1D* mutants, *PHB, PHV,* and *ATHB15* are downregulated. However, *REV* is upregulated and this accounts for adaxialization of leaves but not meristem defects. Again, differential effects of *miR165/166* on Class III HD–ZIP genes may be a consequence of dosage-dependent interactions between microRNA and target and the degree to which expression patterns of these two overlap. This in turn is potentially influenced by regulatory interactions between the different Class III HD–ZIP genes.

## Regulation of Regulators

One gene involved in microRNA-mediated regulation of *PHB* is *ARGONUATE1 (AGO1)*. *AGO1* is a key component of RNA-mediated gene silencing. In plants, *AGO1* binds microRNAs and is sufficient to mediate cleavage of target transcripts [37,38]. Mutations in *ago1* disrupt many aspects of development, including organ dorsoventral patterning. In plants carrying strong *ago1* mutant alleles, lateral organ development is severely affected, leading to a number of disparate interpretations of the phenotype [39–41]. It is possible that both adaxial and abaxial fates are affected by *ago1* such that leaves in severely affected plants have lost polarity. However, in strong *ago1* mutants *PHB* expression is expanded throughout the leaf and weak *ago1* alleles have leaf development defects consistent with adaxialization of the leaf, indicating that one role of *AGO1* is regulation of *PHB* via miRNAs [41].

Another component implicated in microRNA-mediated regulation of *PHB* is *SERRATE (SE)*. Mutations in *SE* have a number of effects on shoot development including time to flowering, meristem size, leaf serrations, and dorsoventral patterning defects [42–45]. In severe mutants, leaves and leaf vasculature are adaxialized. *se* mutants have increased levels of expression of several Class III HD–ZIP genes. In the case of *PHB,* the domain of expression is expanded to the abaxial side of the leaf and the level of *PHB* is increased in the abaxial side of the leaf, similar to the pattern of misexpression in the dominant *phb-1D* mutants. The Class III HD–ZIP genes are likely secondarily affected by *SE* because the level of *miR166* is greatly reduced in *se* mutants [45]. Concomitant with a reduction in *miR166,* the level of the precursor transcript for *miR166* is increased in *se* mutants indicating that *SE* may function in processing miRNA precursor transcripts. *SE* encodes a protein with a domain sharing limited similarity to a zinc finger, although the precise function is still to be determined [44].

Dominant mutations in *PHB* and mutations in *AGO1* and *SE* all result in *PHB* misexpression with increased levels of transcript in the adaxial domain and ectopic expression in the abaxial domain of young leaf primordia. Changes in adaxial and abaxial expression in these backgrounds suggest miRNAs regulating *PHB* expression are expressed throughout early leaf primordia. One report shows *miR165/166* expression throughout the *Arabidopsis* shoot [46]. However two other reports, in *Arabidopsis* and in maize, indicate that these microRNAs are spatially restricted in the shoot apex and may function in patterning [41,47]. A further report examining expression in the embryo indicates dynamic expression throughout development, but in early embryogenesis *miR166* is initially abaxial and at the distal tips of initiating cotyledons, an expression pattern closely complementary to that of the HD–ZIP genes [36]. Resolution of the degree to which *miR165/166* and HD–ZIP target genes overlap may come from analysis of the expression pattern of sensor constructs where a cell-autonomous reporter gene carries the miRNA target binding site [48]. To add to this picture, Class III HD–ZIP tissue-specific expression appears to be directed simply by the promoter [21,22,49], in which case microRNAs may serve to modulate expression levels, particularly at boundaries where regulator and target expression overlap.

The LOB-domain gene *ASYMMETRIC LEAVES2,* which is required in leaf patterning, appears to negatively regulate *miR165*. Levels of *miR165* are further increased in mutants lacking both *ASYMMETRIC LEAVES2* and the RNA silencing pathway gene *RDR6*, an RNA-dependant RNA polymerase [46]. However, regulation of *miR165* by *ASYMMETRIC LEAVES2* and *RDR6* is likely to be indirect as *RDR6* acts in a pathway with three genes, *AGO7, SGS3,* and *DCL4,* known to regulate production of a specific class of small RNAs known as *trans*-acting siRNA [50–55]. Downstream targets of these *trans*-siRNA pathway genes are *ETTIN* and *ARF3,* two AUXIN RESPONSE FACTOR genes required for abaxial fate [50,56,57].

## Linking to Chromatin

Aside from cleavage, microRNA targeting to the *PHB* transcript also influences the methylation status of the *PHB* locus [58]. High levels of methylation are usually associated with transcriptionally inactive chromatin, and, conversely, low methylation levels are typically associated with transcriptionally active chromatin. In wild-type plants the *PHB* locus is methylated at the 3′ end of the gene, although methylation levels are low in meristem-enriched tissues where *PHB* is expressed. The dominant allele, which may no longer effectively bind *miR165/166,* fails to mediate methylation of the *PHB* gene. Thus *miR165/166* may function, directly or indirectly, in transcriptional as well as posttranscriptional regulation of *PHB*. The *PHV* gene is also methylated in the 3′ region indicating Class III HD–ZIP genes as a whole may be subject to multiple levels of regulation. The significance of this regulatory system is yet to be established, but multiple levels of regulation may serve several purposes. Conceivably, microRNA-directed cleavage of transcripts acts as an efficient mechanism for rapid inactivation of transcripts within a cell. Simultaneous or subsequent methylation of the locus would then maintain a stable repressed state in cells where gene expression is no longer required.

## Conclusions

The Class III HD–ZIP genes play multiple, possibly interdependent, roles in plant development. Conservation of these genes and expression patterns throughout land plants, in particular in lower land plant species, highlight a critical role in development of the basic plant body [59]. Spatial, temporal, and quantitative regulation of expression appears to involve a number of mechanisms including posttranscriptional and transcriptional gene silencing mediated by microRNAs. The importance of microRNAs as regulators of this gene family is reflected in conservation of *miR166* and conservation of Class III HD–ZIP gene function in divergent plant species [27,47,60,61]. An additional layer of regulation may involve modulation of function via a sterol-type ligand. Evaluation of the contribution and interplay of these regulatory mechanisms and the degree to which components of regulation are conserved are clearly going to be subjects of much future research.

## Supporting Information

### Accession Numbers

Accession numbers from the Genbank genebank (http://www.ncbi.nlm.nih.gov/Genbank) are for: *PHABULOSA (PHB),* 2G34710; *PHAVOLUTA (PHV),* 1G30490; *ATHB8,* 4G32880; *ATHB15,* 1G52150; and *REVOLUTA (REV),* 5G60690.

## Abbreviations

HD–ZIP: homeodomain–leucine zipper
*phan*: *phantastica*
*se* and *SE*: *SERRATE*
START: sterol/lipid binding domain
